# Deflazacort versus prednisone/prednisolone for maintaining motor function and delaying loss of ambulation: A post HOC analysis from the ACT DMD trial

**DOI:** 10.1002/mus.26191

**Published:** 2018-09-27

**Authors:** Perry B. Shieh, Joseph Mcintosh, Fengbin Jin, Marcio Souza, Gary Elfring, Siva Narayanan, Panayiota Trifillis, Stuart W. Peltz, Craig M. Mcdonald, Basil T. Darras, AND THE ACT DMD STUDY GROUP

**Affiliations:** ^1^ University of California, 300 UCLA Medical Plaza B‐200 Los Angeles Los Angeles, California USA; ^2^ PTC Therapeutics, Inc. 100 Corporate Court, South Plainfield, NJ, South Plainfield New Jersey USA; ^3^ University of California Davis School of Medicine Department of Physical Medicine and Rehabilitation, 4860 Y Street, Suite 3850 Sacramento California USA; ^4^ Boston Children's Hospital, 300 Longwood Avenue Boston Massachusetts USA

**Keywords:** deflazacort, muscular dystrophy, prednisolone, prednisone, walking

## Abstract

*Introduction*: ACT DMD was a 48‐week trial of ataluren for nonsense mutation Duchenne muscular dystrophy (nmDMD). Patients received corticosteroids for ≥6 months at entry and stable regimens throughout study. This post hoc analysis compares efficacy and safety for deflazacort and prednisone/prednisolone in the placebo arm. *Methods*: Patients received deflazacort (*n* = 53) or prednisone/prednisolone (*n* = 61). Endpoints included change from baseline in 6‐minute walk distance (6MWD), timed function tests, estimated age at loss of ambulation (extrapolated from 6MWD). *Results*: Mean changes in 6MWD were ‐39.0 m (deflazacort; 95% confidence limit [CL], ‐68.85, ‐9.17) and ‐70.6 m (prednisone/prednisolone; 95% CL, ‐97.16, ‐44.02). Mean changes in 4‐stair climb were 3.79 s (deflazacort; 95% CL, 1.54, 6.03) and 6.67 s (prednisone/prednisolone; 95% CL, 4.69, 8.64). *Conclusions*: This analysis, limited by its post hoc nature, suggests greater preservation of 6MWD and 4‐stair climb with deflazacort vs. prednisone/prednisolone. A head‐to‐head comparison will better define these differences. *Muscle Nerve* **58**: 639–645, 2018

**Abbreviations**
6MWD: 6‐minute walk distance
6MWT: 6‐minute walk test
ACT DMD: Ataluren Confirmatory Trial in Duchenne muscular dystrophy
BMI: body mass index
CL: confidence limit
CINRG‐DNHS: Cooperative International Neuromuscular Research Group‐Duchenne Natural History Study
DMD: Duchenne muscular dystrophy
FOR‐DMD: Finding the Optimum Regimen for Duchenne muscular dystrophy
HRQoL: health‐related quality of life
ITT: intent‐to‐treat
LoA: loss of ambulation
LS: least square
MMRM: mixed model repeated measures
nm: nonsense mutation
NSAA: North Start Ambulatory Assessment
PODCI: Pediatric Outcomes Data Collection Instrument
SEA: severe adverse event
TEAE: treatment‐emergent adverse event
TFT: Timed Function Test

Duchenne muscular dystrophy, a rare, irreversible, X‐linked disorder, results in a progressive decline in muscle function and when left untreated, leads to loss of ambulation (LoA) by age 10–12 years and death from cardiac or respiratory failure by the late teens to approximately 25 years of age.^1^, ^2^, ^3^, ^4^, ^5^ The current standard of care for DMD includes corticosteroid therapy with prednisone, prednisolone, or deflazacort,^2^ with accumulating evidence suggesting that these agents can slow the decline in muscle strength and motor function, delay LoA, possibly decrease the development of scoliosis requiring operative management, and slow the loss of upper limb function and the rate of pulmonary function decline.^5^, ^6^, ^7^, ^8^

Deflazacort is a synthetic corticosteroid characterized by the insertion of a fused methyl‐oxazoline ring in the chemical structure of prednisone, with a long duration of action.^9^ A phase 3, randomized, double‐blind, placebo‐controlled, 12‐week trial of 2 doses of deflazacort (0.9 mg/kg/d and 1.2 mg/kg/d) or prednisone dosed at 0.75 mg/kg/d showed that each dosage level of deflazacort or prednisone increased muscle strength from baseline compared with placebo in just 12 weeks.^10^

The objective of this post hoc analysis from a previously published study^11^ is to compare the efficacy and safety of deflazacort and prednisone/prednisolone in slowing DMD disease progression as it relates to physical functioning and the potential for delay in LoA.

## MATERIALS AND METHODS

### Study Design

The Ataluren Confirmatory Trial in patients with nonsense mutation DMD (nmDMD) (ACT DMD, NCT01826487) study was a randomized, double‐blind, placebo‐controlled 48‐week trial that evaluated ataluren's treatment effect in stabilizing motor function and delaying disease progression in patients with nmDMD.^11^

All patients enrolled in this study had been receiving corticosteroid therapy (deflazacort or prednisone/prednisolone) for ≥6 months at study entry, had no clinically significant change in dosage or dosing regimen for ≥3 months before study entry, and were expected to maintain a stable dose and regimen during the study.^11^ Dosing alterations necessitated by changes in body weight were allowed. The placebo arm consisted of 114 patients in the intent‐to‐treat (ITT) population, 53 of whom received deflazacort and 61 of whom received prednisone/prednisolone at entry and throughout the study. The size of these subgroups made a comparison of findings with these corticosteroids feasible. The present analysis was conducted using data from the placebo arm of the ACT DMD trial.

### Patients

This phase 3 study enrolled ambulatory male patients with phenotypic and genotypic confirmation of nmDMD aged 7–16 years. Inclusion and exclusion criteria were as described previously.^11^

Parents or guardians provided written informed consent, and patients provided written assent when appropriate. The trial and any changes to the protocol were approved by the local regulatory authorities and the institutional review board of each site. The trial was done in accordance with the Declaration of Helsinki (2000) and the principles of Good Clinical Practice, according to the International Council for Harmonisation tripartite guideline.

### Efficacy Endpoints

Efficacy assessments were conducted every 8 weeks at clinic visits.^12^ The primary endpoint assessed the ability of treatment to slow the progression of disease and was evaluated by the change from baseline to Week 48 in 6‐minute walk distance (6MWD). The secondary endpoints evaluated the effect of treatment on proximal muscle function using Timed Function Tests (TFTs; 4‐stair climb/descent, rise from supine, 10 m walk/run). Exploratory assessments consisted of the North Star Ambulatory Assessment (NSAA) and Pediatric Outcomes Data Collection Instrument (PODCI) domains of Transfer/Basic Mobility and Sports/Physical Functions.^11^ The NSAA is a validated, DMD‐specific scale shown to be sensitive to change. It evaluates 17 functional abilities relevant to ambulant patients and scores these as 0, unable to perform; 1, performs with difficulty; or 2, able to perform for a total score from 0 (worst) to 34 (best).^12^, ^13^, ^14^, ^15^ The PODCI evaluates health‐related quality‐of‐life (HRQoL) outcomes with each domain scored from 0 (worst) to 100 (best).^11^, ^16^ The PODCI was developed to evaluate functional outcomes of musculoskeletal health in children and adolescents and has been shown to be reliable and have valid construct and sensitivity to change.^16^

### Safety Endpoints

Safety assessments included type, frequency, severity, timing, and relationship to study drug of adverse events that were recorded throughout the study, laboratory abnormalities and changes in vital signs assessed every 8 weeks, and findings from physical examinations at 24 and 48 weeks.^11^

### Statistical Analyses

In this analysis, corticosteroid efficacy data were analyzed for the ITT population, consisting of all patients who were randomized to placebo and who had a valid 6MWD value at baseline and ≥1 valid post‐baseline 6MWD assessment.^12^ Comparisons were made according to corticosteroid treatment at baseline (deflazacort vs. prednisone/prednisolone).

The analysis used mixed model repeated measures (MMRM) with multiple imputation for missing data values using SAS programs Proc MI to create multiple imputed datasets and Proc MIANALYZE to combine the inferences from each dataset into a single one. The model included the following factors: age (<9 years, ≥9 years), baseline 6MWD (<350 m, ≥350 m), duration of prior corticosteroid (≥6 months to <12 months, ≥12 months), corticosteroid subgroup (deflazacort, prednisone/prednisolone), visit (as a class variable), interaction of visit with previously mentioned factors, baseline value as a covariate, and interaction of visit with baseline value. Least square means for changes in 6MWD and TFTs from baseline to study end and treatment differences were calculated. For patients who were unable to perform the 6‐minute walk test (6MWT) at a study visit, a value of 0 m was assigned. For patients who were unable to perform a TFT and for those whose performance was >30 s, a value of 30 s was assigned. Standardized t‐statistics were used to allow for the reporting of multiple endpoints (6MWD, TFTs, and the NSAA and PODCI scores) on the same scale on a Forest plot. Values were calculated by dividing the point estimate for the difference between deflazacort vs. prednisone/prednisolone in mean change at Week 48, and the 95% confidence limits (CL), each by the standard error. For endpoints where improved function is represented by a negative value (i.e., TFTs), the signs of the values were reversed.

For determination of LoA, the 48‐week changes from baseline in 6MWD observed in patients taking deflazacort or prednisone/prednisolone were annualized. For patients who did not reach LoA by Week 48, linear extrapolation was used to estimate the number of years it would take to reach LoA (a 6MWD of 0 m) according to the method of Clayton and colleagues.^17^ This model, however, does not consider the potential nonlinear decline in 6MWD in older patients with DMD.^18^

The duration of exposure to either corticosteroid before study entry was determined using the following assumptions: (1) if end date of corticosteroid therapy before study entry was missing, the end date was noted as the start date of placebo; (2) if end date of corticosteroid therapy before study entry was on or after the start date of placebo, the end of corticosteroid therapy was noted as the start date of placebo.

Safety was analyzed in the as‐treated population, which consisted of all randomized patients who received any study treatment.^12^

## RESULTS

### Patients

A total of 115 patients enrolled in the ACT DMD trial were randomized to placebo.^11^ The ITT population for the placebo arm consisted of 114 patients, with 53 patients receiving deflazacort and 61 receiving prednisone/prednisolone. One patient with a valid post‐baseline 6MWD was discontinued because gene sequencing did not confirm the presence of a nonsense mutation in the dystrophin gene.^11^

The characteristics (Table 1 and Supplementary Table S1, which is available online) of the patients taking either deflazacort or prednisone/prednisolone were well balanced at baseline. These patients had a mean age of 9 years, and most of these patients had been receiving corticosteroid therapy for ≥12 months before entering the study.

**Table 1:** Baseline patient demographics (ITT population)

Characteristic	Deflazacort (*n* = 53)	Prednisone/prednisolone (*n* = 61)	Total (*n* = 114)
Age, y			
Mean (SD)	9.2 (1.7)	8.8 (1.6)	9.0 (1.7)
Range	7,14	7,13	7,14
Age group, *n* (%)			
<9 y	23 (43.4)	30 (49.2)	53 (46.5)
≥9 y	30 (56.6)	31 (50.8)	61 (53.5)
Race, *n* (%)			
White	46 (86.8)	40 (63.9)	85 (74.6)
Black/African American	0 (0.0)	1 (1.6)	1 (0.9)
Asian	4 (7.5)	2 (3.3)	6 (5.3)
Hispanic	3 (5.7)	5 (8.2)	8 (7.0)
Other	0 (0.0)	4 (6.6)	4 (3.5)
Missing	0 (0.0)	10 (16.4)	10 (8.8)
Weight, kg			
Mean (SD)	30.9 (11.9)	30.5 (9.2)	30.7 (10.5)
Range	18.1, 68.0	18.2, 59.8	18.1, 68.0
Height, cm			
Mean (SD)	127.010.6)	125.7 (10.4)	126.3 (10.4)
Range	106.7, 148.7	101.8, 151.0	101.8, 151.0
BMI, kg/m^2^			
Mean (SD)	18.6 (4.70)	19.0 (3.5)	18.9 (4.1)
Range	13.0, 36.0	13.1, 27.1	13.0, 36.0
Corticosteroid use prior to baseline, *n* (%)			
6 to <12 months	7 (13.2)	11 (18.0)	18 (15.8)
≥12 months	46 (86.8)	50 (82.0)	96 (84.2)

*SD, standard deviation*.

There was no significant difference in the duration of exposure to deflazacort or prednisone before study entry. Patients treated with deflazacort had a mean exposure of 1062 days (range, 189–2743 days), and those treated with prednisone/prednisolone had a mean exposure of 1081 days (range, 124–2698 days; *P* = 0.86, *t*‐test) before study entry.

Dosing data in mg/kg/day were available for 110 patients. For the other 4 patients in the ITT population, the dosing record described liquid volume or number of drops of suspension without drug concentration, and, therefore, these patients were excluded from the summary of dosing. In the prednisone/prednisolone subgroup, dosing regimens consisted of daily (64.4%), every other day (16.9%), 10 days on followed by 10 days off (10.2%), and high‐dose weekend (8.5%). In the deflazacort subgroup, dosing regimens consisted of daily (84.3%), every other day (13.7%), and twice a day (2.0%). Among patients on a daily dosing regimen, the mean dose was relatively lower for prednisone/prednisolone (0.515 mg/kg/day, recommended 0.75 mg/kg/day, 69% of recommended) than deflazacort (0.695 mg/kg/day, recommended 0.9 mg/kg/day, 77% of recommended).

### Physical Functioning

**Figure 1:**
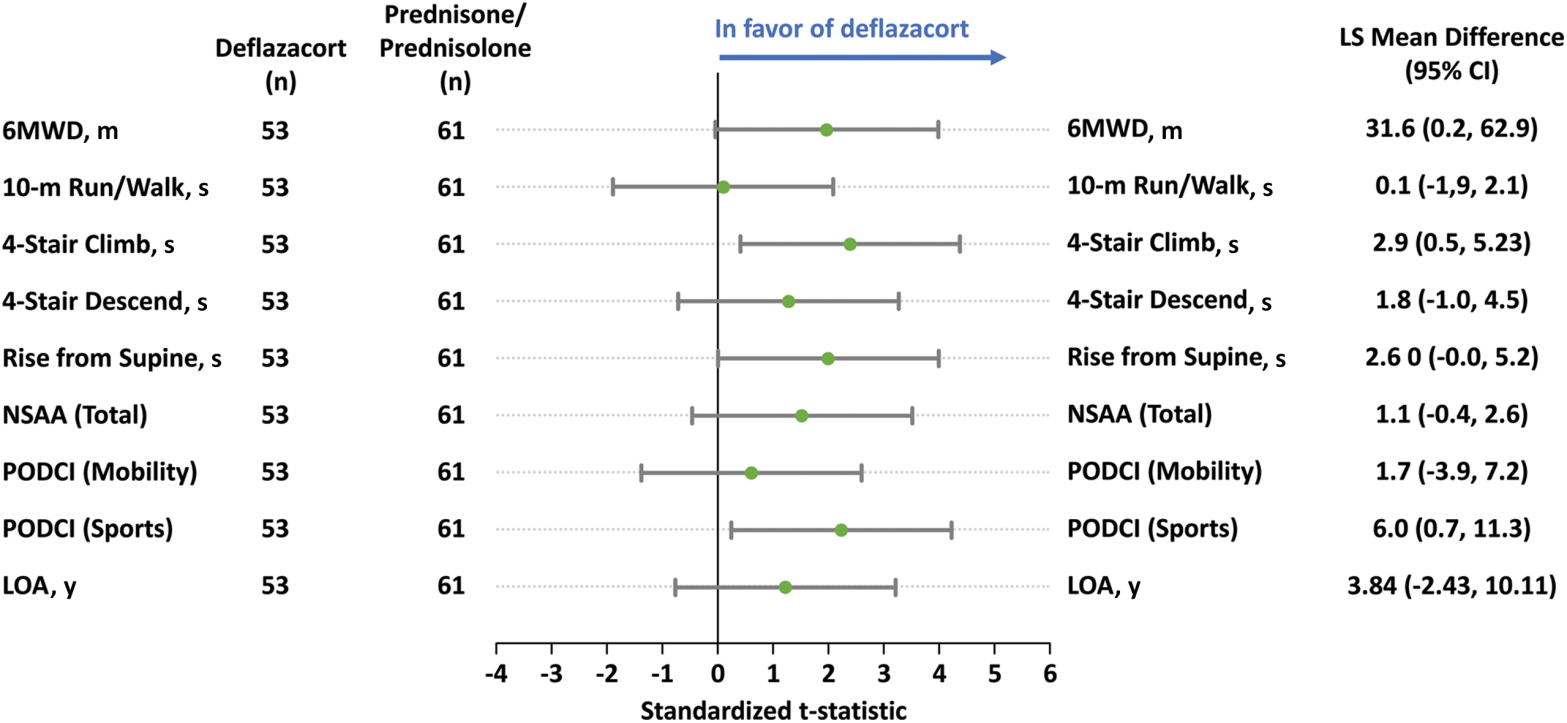
Least‐square mean changes and t‐statistics from baseline to week 48 in assessments of physical functioning and HRQoL (ITT) for deflazacort versus prednisone/prednisolone.

Patients treated with deflazacort had notably less decline from baseline in 6MWD at Week 48 than those treated with prednisone/prednisolone (Table 2; Fig. 1). The extrapolated time to loss of ambulation when using a linear model was 8.58 years for deflazacort and 4.74 years with prednisone/prednisolone, a noteworthy difference.

**Table 2:** Least squares mean change (95% CL) from baseline to week 48 in assessments of physical functioning and HRQoL (ITT)

Endpoint Δ (SE) (95% CL)	Deflazacort (*n* = 53)	Prednisone/prednisolone (*n* = 61)	Difference (95% CL)
6MWD, m	−39.01 (15.05)	−70.59 (13.40)	31.6
	(−68.85, −9.17)	(−97.16, −44.02)	(0.22, 62.94)
TFTs, s			
4‐Stair climb	3.79 (1.13)	6.67 (1.0)	−2.88
	(1.54, 6.03)	(4.69, 8.64)	(−5.27, −0.48)
4‐Stair descent	3.89 (1.29)	5.66 (1.12)	−1.77
	(1.33, 6.45)	(3.43, 7.89)	(−4.51, 0.98)
Rise from supine	4.50 (1.24)	7.10 (1.13)	−2.60
	(2.05, 6.95)	(4.86, 9.34)	(−5.20, 0.01)
10‐m walk/run	3.16 (0.93)	3.25 (0.85)	−0.09
	(1.32, 5.00)	(1.56, 4.94)	(−2.07, 1.89)
NSAA total score	−3.39 (0.70)	−4.53 (0.66)	1.14
	(−4.78, −2.01)	(−5.83, −3.23)	(−0.36, 2.64)
PODCI			
Sports/Physical Functioning	−4.80 (2.49)	−10.76 (2.25)	5.96
	(−9.73, 0.13)	(−15.21, −6.31)	(0.65, 11.28)
Transfers/Basic/Mobility	−7.53 (2.62)	−9.20 (2.34)	1.67
	(−12.72, −2.35)	(−13.84, −4.57)	(−3.87, 7.21)

*Δ, mean change*.

Results for the 4‐stair climb showed that the LS mean increase in time from baseline to Week 48 with deflazacort was approximately half of that with prednisone/prednisolone, (Table 2; Fig. 1). For the other TFTs (4‐stair descend, rise from supine, 10‐m walk/run) and the NSAA total score, LS mean changes also notably favored deflazacort (Table 2; Fig. 1).

The mean decline in the domain of Sports/Physical Function in HRQoL for the patients receiving deflazacort was less than that for the patients receiving prednisone/prednisolone (Table 2). The treatment difference for the Transfers/Basic Mobility domain of the PODCI also favored deflazacort.

### Safety

The safety profiles for deflazacort and prednisone/prednisolone were generally comparable. No significant differences were noted. Table 3 lists treatment‐emergent adverse events (TEAEs) occurring in either subgroup with an incidence >5%. The incidence of the following TEAEs was numerically lower for the deflazacort subgroup than for the prednisone/prednisolone subgroup: nasopharyngitis, abdominal pain, back pain, pyrexia, and upper respiratory tract infection. The remaining TEAEs were similar between the 2 subgroups, with none being markedly lower in the prednisone/prednisolone subgroup.

**Table 3:** Most common* TEAEs (as‐treated population)

TEAE, *n* (%)	Deflazacort (*n* = 53)	Prednisone/Prednisolone (*n* = 62)
Pain in abdomen (including upper abdomen)	0 (0)	18 (29)
Nasopharyngitis	6 (11)	17 (27)
Headache	10 (19)	11 (18)
Vomiting	10 (19)	11 (18)
Fall	8 (15)	12 (19)
Pain in extremity	6 (11)	8 (13)
Cough	5 (9)	8 (13)
Pyrexia	4 (8)	8 (13)
Constipation	4 (8)	6 (10)
Back pain	2 (4)	6 (10)
Upper respiratory tract infection	0 (0)	6 (10)
Diarrhea	5 (9)	5 (8)
Ligament sprain	3 (6)	4 (6)
Nausea	3 (6)	4 (6)
Oropharyngeal pain	2 (4)	4 (6)

* *Incidence of ≥5% in either subgroup*.

Patients in the placebo arm receiving deflazacort had numerically smaller increases in weight, height, and BMI during the study than patients receiving prednisone/prednisolone (Table 4).

**Table 4:** Mean changes from baseline in weight, height, and BMI at week 48

Measurement	Deflazacort (*n* = 53)	Prednisone/prednisolone (*n* = 62)
Weight, kg		
*n*	50	59
Mean change (SD)	3.9 (2.6)	4.6 (3.2)
95% CL	3.2, 4.6	3.8, 5.4
Median	3.8	3.8
Height, cm		
*n*	50	59
Mean change (SD)	3.2 (2.0)	3.9 (1.9)
95% CL	2.7, 3.8	3.4, 4.4
Median	3.0	4.0
BMI, kg/m^3^		
*n*	50	59
Mean change (SD)	1.3 (1.3)	1.6 (1.5)
95% CL	1.0, 1.7	1.2, 1.9
Median	1.3	1.4

*CI, confidence limit; SD, standard deviation*.

Most TEAEs were mild to moderate in severity and included a mild T12 vertebral fracture in a patient treated with deflazacort. Severe AEs consisted of 1 case each of back pain and vomiting in the deflazacort subgroup and 1 case each of gait disturbance and muscular weakness in the prednisone/prednisolone subgroup. A total of 4 patients had serious adverse events (SAEs). In the deflazacort subgroup, the 3 SAEs included myocarditis, abnormal hepatic function test, and femur and lower limb fractures. The fourth SAE, which occurred in the prednisone/prednisolone subgroup, was gastroenteritis. One patient receiving deflazacort and none of the patients receiving prednisone/prednisolone discontinued the trial due to loss of ambulation.

## DISCUSSION

This post hoc analysis of the placebo arm of the ACT DMD study demonstrated less mean decline from baseline to week 48 in 6MWD, lower mean declines from baseline in the 4‐stair climb and the PODCI domain of Sports/Physical Function in HRQoL, and a longer estimated duration of ambulation in patients treated with deflazacort than with prednisone/prednisolone. Current practice guidelines recommend use of corticosteroids for their benefits on muscle strength and motor function and delay in LoA in patients with DMD.^2^ Although their mechanism of action in DMD is not completely understood, the anti‐inflammatory properties of these agents have been implicated along with stabilizing effects on muscle fiber membranes, inhibition of muscle proteolysis, stimulation of myoblast proliferation, and differential gene regulation.^5^, ^6^, ^7^

The 6MWT is a globally accepted assessment of endurance and muscle function in patients with neuromuscular diseases who are capable of ambulation.^19^, ^20^ The treatment difference observed in this study between deflazacort and prednisone/prednisolone for the 6MWT was associated with a 95% CL with a lower limit that approached 0. However, the magnitude of the difference was substantially larger than the published standards for a clinically meaningful difference. A 6MWD change of 10 to 20 m is clinically meaningful across a wide range of ambulatory patients with DMD.^21^ The treatment difference observed in this post hoc analysis for the 4‐stair climb also meets published standards for a clinically meaningful minimal difference of 1.5 s.^22^

Prolonging ambulation is a key goal of treatment.^2^ Loss of ambulation constitutes a substantial disability for DMD patients and it marks the beginning of a more severe stage of the disease. Delaying LoA has been correlated with delaying the time to loss of subsequent disease milestones such as decline in respiratory function.^8^, ^23^

The finding for LoA in this post hoc analysis is consistent with data from the large (N = 340), observational, Cooperative International Neuromuscular Research Group‐Duchenne Natural History Study (CINRG‐DNHS), in which deflazacort further delayed LoA by nearly 3 years compared with prednisone/prednisolone (daily regimen) 24 and those from a more recently published, long‐term, prospective, cohort study from CINRG (N = 440) that showed further delays of 2.7 years in LoA with deflazacort compared with prednisone/prednisolone.^8^

The DMD Care Considerations guidelines recommend daily dosing of patients with DMD with corticosteroids unless the therapy is not well tolerated, in which case an alternative regimen may be used.^2^ The most common corticosteroid dosing regimen for patients in the placebo arm of the ACT DMD trial was once daily. There was a higher percentage of patients on daily deflazacort therapy compared with patients receiving daily prednisone/prednisolone. A possible reason for this observation may be that daily therapy with deflazacort was better tolerated in these patients than daily therapy with prednisone/prednisolone. This reasoning may also be supported by the fact that the daily mean dose was higher relative to the recommended dose for deflazacort than for prednisone/prednisolone. The corticosteroid regimens were determined by the patients' physicians before the study and are thus representative of real‐world use of these agents.

Deflazacort treatment in this post hoc analysis was associated with slightly lower mean growth over 48 weeks than treatment with prednisone/prednisolone. Corticosteroid effects on growth may influence delay of LoA, possibly by conferring biomechanical advantages for walking.^25^, ^26^ A prospective, longitudinal, multicenter study in the United Kingdom observed a possible link between shorter stature and delayed LoA in patients with DMD receiving corticosteroids.^15^ An analysis of anthropomorphic effects found that patients treated with deflazacort had significantly shorter stature (*P* < 0.002) than those treated with prednisone over a 52‐week follow‐up.^2^, ^6^

Weight gain may contribute to disability in patients with DMD by increasing the load on deteriorating muscles. Corticosteroid therapy has been associated with weight gain, and some studies in patients with DMD have noted less weight gain with deflazacort than with prednisone, including an analysis of anthropomorphic effects of corticosteroid therapy in DMD^26^ and a systematic review of clinical trials of corticosteroid treatment in patients with DMD.^7^ Consistent with these studies, patients receiving deflazacort in this post hoc analysis had a lower mean increase in weight than those receiving prednisone/prednisolone and a smaller mean increase in BMI.

Other adverse effects of corticosteroid therapy in DMD clinical trials include behavioral abnormalities, cushingoid appearance, excessive hair growth, and cataracts.^6^, ^7^ Deflazacort has been associated with a lower incidence of these events, with the exception of cataracts.^6^ None of these events were reported for patients in the placebo arm of the ACT DMD trial.

This analysis has several limitations, including its post hoc nature and the fact that the ACT DMD trial was not powered to detect specific treatment differences in these subgroups of the placebo arm. For rare diseases, retrospective analyses can provide insights and contribute to the body of data on medical interventions. Because deflazacort was not commercially available in the United States until 2017,^27^ a potential confounder of this post hoc analysis is that in the United States, patients with DMD who were treated with deflazacort could have been from families of high socioeconomic means and may have been receiving better supportive care, which may have affected the outcomes of this analysis. However, of the 230 total patients in ACT DMD, 162 were from outside the United States (70.4%). The placebo arm, which was the source of the data for this post hoc analysis, had 115 total patients in the as‐treated population. Eighty‐three of the 115 total patients in the placebo arm were from outside the United States, (72.2%), with 37 of the 83 treated with deflazacort while 46 received prednisone. Only 32 patients of the 115 total patients were from the United States, with 16 of the 32 treated with deflazacort while the remaining 16 patients were treated with prednisone. The treatment benefits observed in patients who received deflazacort in this study are, therefore, not likely attributable to socioeconomic status, as most of the study subjects were from outside the United States, where deflazacort was and remains readily available as a generic drug. Additionally, the inclusion criterion of a 6MWD ≤ 80% of predicted for the ACT DMD trial made it possible to enroll patients with a wide range of disease severity, which is representative of patients in the real world. As mentioned previously, the baseline characteristics of the patients from the deflazacort and prednisone/prednisolone groups were comparable.

Additional evidence comparing the benefits of deflazacort and prednisone/prednisolone in patients with DMD may become available from an analysis of data from the placebo arm of a 48‐week, phase 3, randomized, double‐blind, placebo‐controlled trial that evaluated the efficacy and safety of treatment with tadalafil for prolonging ambulation. This study had the same requirements for corticosteroid therapy before and during the trial as those in the ACT DMD trial. The ongoing international, multicenter, double‐blind, parallel‐group, 3‐year Finding Optimum Regimen for DMD (FOR‐DMD) trial (NCT01603407) 28 comparing daily and intermittent regimens of prednisone to daily deflazacort in approximately 300 patients with DMD may further augment the understanding of benefits associated with these 2 steroid treatments and 2 regimens (daily versus intermittent). However, it is noteworthy that this trial has focused on a younger cohort of patients with DMD ages 4 to 6 years who are not necessarily expected to experience functional deterioration while on steroids.

In conclusion, these findings suggest that deflazacort therapy may confer benefits as compared with prednisone/prednisolone in patients with DMD, including less decline in distance walked, less time needed for 4‐stair climbing, and greater delay in LoA. The availability of treatments that have the potential to alter the natural history of DMD supports the need for early diagnosis in patients with this disease.

## Supporting information

Supporting Information
