# Supplementary material for: Deflazacort versus prednisone/prednisolone for maintaining motor function and delaying loss of ambulation: A post HOC analysis from the ACT DMD trial
Source: Muscle Nerve. 2018 Sep 27;58(5):639–45. doi: 10.1002/mus.26191 (PMC6767037; doi:10.1002/mus.26191)
Supplement: Supplementary file 1 — Supporting Information [file MUS-58-639-s001.docx]

**Supplemental Data**

**Table S1.** Baseline 6-minute walk distance and timed function test data
(intent-to-treat population)

| Characteristic | Deflazacort (n = 53) | | Prednisone/ prednisolone  (n = 61) | Total  (n = 114) |  |  |  |  |
| --- | --- | --- | --- | --- | --- | --- | --- | --- |
| 6MWD, m  n  Mean  Range | 53  361.3 (87.7) 159.5, 526.0 | | 61  365.5 (76.0) 142.5, 518.5 | 114  363.5 (81.3)  142.5, 526.0 |  |  |  |  |
| 4-stair climb, s  n  Mean (SD)  Range | 51 6.4 (6.9) 1.1, 30.0 | | 60 6.4 (4.3) 1.4, 22.7 | 111 6.4 (5.7) 1.1, 30.0 |  |  |  |  |
| 4-stair descent, s   n  Mean (SD)  Range | 49 4.5 (4.3) 1.2, 30.0 | | 59 5.0 (4.7) 1.1, 30.0 | 108 4.8 (4.5) 1.1, 30.0 |  |  |  |  |
| Rise from supine, s   n  Mean (SD)  Range | | 50 8.7 (7.7) 1.9, 30.0 | 60 10.4 (7.9) 2.1, 30.0 | 110 9.6 (7.8) 1.9, 30.0 |  |  |  |  |
| Continued  **Table S1.** Baseline 6-minute walk distance and timed function test data  (intent-to-treat population) continued | | | | | |  |  |  |
| 10-m Walk/run   n  Mean  Range | | 53  6.6 (3.2) 3.2, 17.0 | 61  7.00 (2.6) 3.7, 16.0 | 114  6.80 (2.9) 3.2, 17.0 |  |  |  |  |
| Total NSAA  n  Mean (SD)  Range | | 53  23.0 (9.0) 4, 34 | 61 20.9 (7.0) 7, 32 | 114 21.9 (8.0) 4, 34 |  |  |  |  |
| PODCI | |  |  |  |  |  |  |  |
| Transfers/Basic Mobility  n  Mean (SD)  Range | | 53  80.1 (16.3) 42, 100 | 61  82.5 (15.4)  32, 100 | 114  81.4 (15.8)  32,100 |  |  |  |  |
| Sports/Physical Functioning  n  Mean (SD)  Range | | 53 54.3 (22.9) 8, 97 | 61 57.4 (19.2) 18, 95 | 114  56.0 (21.0)  -8, 97 |  |  |  |  |

6MWD = 6-minute walk distance; NSAA = North Star Ambulatory Assessment;
PODCI = Pediatric Outcomes Data Collection Instrument; SD = standard deviation
